# Role of ultrasound guided transversus abdominis plane block as a component of multimodal analgesic regimen for lower segment caesarean section: a randomized double blind clinical study

**DOI:** 10.1186/s12871-018-0512-x

**Published:** 2018-05-14

**Authors:** Ashok Jadon, Priyanka Jain, Swastika Chakraborty, Mayur Motaka, Sudhansu Sekhar Parida, Neelam Sinha, Amit Agrawal, Asit Kumar Pati

**Affiliations:** Department of Anaesthesia and Pain Relief Service, Tata Motors Hospital, Telco Colony, Jamshedpur, Jharkhand 831004 India

**Keywords:** Transversus abdominis plane block, Caesarean section, Spinal anaesthesia, Post-operative pain, Pain management

## Abstract

**Background:**

While opioids are the mainstay for post-operative analgesia after lower segment caesarean section, they are associated with various untoward effects. Ultrasound guided transversus abdominis plane (TAP) block has been postulated to provide effective analgesia for caesarean section. We evaluated the analgesic efficacy of this block for post caesarean analgesia in a randomised controlled trial.

**Methods:**

One hundred thirty-nine mothers undergoing caesarean delivery were randomised to receive TAP block with either 20 ml 0.375% ropivacaine or 20 ml saline after obtaining informed consent. All the subjects received a standard spinal anaesthetic and diclofenac was administered for post-operative pain. Breakthrough pain was treated with tramadol. Post-operatively, all the subjects were assessed at 0, 2, 4, 6, 8, 10, 12, 18 & 24 h. The primary outcome was the time to first analgesic request. The secondary measures of outcome were pain, nausea, sedation, number of doses of tramadol administered and satisfaction with the pain management.

**Results:**

The median (interquartile range) time to first analgesic request was prolonged in the TAP group compared to the control group (*p* < 0.0001); 11 h (8,12) and 4 h (2.5,6) respectively. The median (interquartile range) number of doses of tramadol consumed in the TAP group was 0 (0,1) compared to 2 (1,2) in the control group (*p* < 0.0001). At all points in the study, pain scores both at rest and on movement were lower in the study group (*p* < 0.0001). Maternal satisfaction with pain relief was also higher in the study group (*p* 0.0002). One subject in the TAP group had convulsions following injection of local anaesthetic solution. She was managed conservatively with supportive treatment following which she recovered.

**Conclusion:**

TAP block reduces pain, prolongs the duration of analgesia and decreases supplemental opioid consumption when used for multimodal analgesia for pain relief after caesarean section. However, the risk of local anaesthetic systemic toxicity remains unknown with this block. Hence larger safety trials and measures to limit this complication need to be ascertained.

**Trial registration:**

The trial was registered with the Clinical Trial Registry of India (CTRI/2017/03/008194) on 23/03/2017 (trial registered retrospectively).

## Background

Lower segment caesarean section (LSCS) is a major surgical procedure with substantial post-operative pain [1]. Good control of pain following LSCS is essential to facilitate early mobilisation and to enable adequate care of the new born. Achieving good pain relief is challenging because of the altered physiology and of the possibility of transmission of drugs through breast milk. Although a variety of choices of drugs and routes of administration are available, we are yet to achieve a safe and effective method of pain control after LSCS.

Conventional analgesic regimens use opioids administered through systemic and/or neuraxial routes. Neuraxial methods are effective and safe, but need to be performed by an experienced person and require very close monitoring [2]. Opioids can also be delivered using intravenous or epidural patient controlled analgesia (PCA). PCA allows patients to have control over their pain management and hence improves their satisfaction with the therapy [3]. However, unwanted effects like sedation, nausea and vomiting, pruritus and occasionally respiratory depression remain the major drawbacks of opioids [4]. Secretion into breast milk is the additional concern in this population [2]. Non-steroidal anti-inflammatory drugs (NSAIDs) and paracetamol can only supplement other modes of analgesia and are not sufficient on their own [2].

Given these issues, peripheral nerve block techniques like transversus abdominis plane (TAP) block were introduced as an effective component of multimodal analgesia after caesarean delivery [5]. These techniques not only reduced pain quite successfully but also eliminated some of the problems associated with the use of systemic opioids or central neuraxial blocks [6, 7]. Ultrasound guided transversus abdominis plane (TAP) block is one such effective method of providing post-operative analgesia for lower abdominal surgeries [8].

The purpose of this randomised study was to evaluate the efficacy of TAP block for post LSCS pain specifically targeting the Indian population. We assessed the role of this block as a component of a multimodal analgesic regimen that excludes intrathecal morphine.

## Methods

The trial was approved by the Ethics and Scientific committee of the institution and registered with the Clinical Trial Registry of India (CTRI/2017/03/008194). A total of 139 mothers aged > 18 years with no major systemic disease, who were scheduled for caesarean section under spinal anaesthesia were enrolled into the study after obtaining written informed consent. Exclusion criteria were a history of drug allergy or local anaesthetic toxicity, BMI (body mass index) > 35 kg/m^2^ and pregnancy weight < 50 kg (to limit maximum ropivacaine dose to 3 mg/kg), contraindications to regional anaesthesia (bleeding diathesis, infection at the site of block and peripheral neuropathy), severe medical conditions such as severe pre-eclampsia and eclampsia and patients who had intra-operative complications like post-partum haemorrhage.

The subjects were randomly allocated into treatment and control groups using a computer generated sequence of random numbers. The group sequence was concealed in sealed opaque envelopes which were opened only after obtaining informed consent. The injectate syringes containing either 40 ml saline or 40 ml 0.375% ropivacaine were prepared by an anaesthesiologist not involved in the study. The anaesthesiologists, the subjects and the post-operative care providers were blinded to the group assignment.

As per usual hospital practice, pre anaesthetic evaluation was done and metoclopramide (10 mg) and ranitidine (50 mg) were given intravenously as premedications 1 h before surgery. All the study subjects received a standard spinal anaesthetic consisting of 11-12.5 mg of 0.5% hyperbaric bupivacaine. Heart rate, blood pressure & pulse oximetry was monitored in the operating room. All the subjects received 75 mg diclofenac IV before the completion of the surgery.

At the end of the surgery, bilateral US (ultrasound) guided TAP block was performed by one of the investigators using either 20 ml of 0.375% ropivacaine (obtained by mixing 10 ml of 0.75% ropivacaine with 10 ml of normal saline) or 20 ml saline on each side. The procedure was performed using aseptic technique (gown, gloves, facemask and protective sheath for the ultrasound probe). After preparing the skin with antiseptic solution, a linear high frequency ultrasound probe (6-13 MHz, Sonosite M-Turbo©) was placed transversely on the anterolateral abdominal wall between the iliac crest and the costal margin. Under US guidance, the three layers of muscles -external oblique, the internal oblique, and the transversus abdominis were identified. A 21-gauge, 100-mm needle attached with flexible tubing to a syringe filled with saline was used to perform the block. The needle was then introduced through the skin anteriorly in the plane of the ultrasound beam and advanced into the fascial plane between the internal oblique and transversus abdominis muscles with its tip lying in the mid axillary line. To assist with identifying these structures, the probe was moved anteriorly to the rectus sheath and the fascial planes followed laterally. The final position of the probe was to be no further anterior than the anterior axillary line. If satisfactory views were not obtained, the TAP block was not performed. Hydro dissection with saline (2-5 ml) was used to separate the fascial layers. After aspiration to exclude inadvertent vascular puncture, a test dose of 1-2 ml of the drug was injected to confirm needle placement. After a negative test dose, 20 ml of the study solution was injected while closely observing for signs of toxicity (tinnitus, perioral numbness, metallic taste in mouth, slurring of speech and mental status changes). TAP block was performed in a similar fashion on the opposite side.

After completion of the procedure, patients were shifted to the post anaesthesia care unit (PACU) before transferring them to the ward. Both the groups received a standard post-operative analgesic regimen consisting of 75 mg of IV diclofenac every 12 h and 50 mg IV tramadol on demand for breakthrough pain.

All the subjects were assessed at 0, 2, 4, 6, 8, 10, 12, 18 & 24 h after surgery for pain at rest & on movement, nausea and sedation. All the subjects were asked to rate their pain at rest and on movement using a visual analogue scale (VAS) with ‘0’ representing no pain and ‘10’ being the worst imaginable pain. Supplemental analgesia (50 mg tramadol IV) was administered if VAS > 4 on movement or if the mother demanded for it. The time to first analgesic request was noted in all the subjects. If no supplemental analgesia was given within 12 h of surgery, regular dose of diclofenac was administered and the duration of analgesia was considered as 12 h. The severity of nausea was measured according to a 4-point rating score (0- absent, 1- mild, 2- moderate, and 3- severe or vomiting). 4 mg ondansetron IV was offered to subjects who complained of nausea or vomiting. Subjects requiring ≥2 doses were given ondansetron round the clock (4 mg thrice daily). A 4-point scale was used to assess sedation (1- fully awake; 2- somnolent, responds to verbal stimuli; 3- somnolent, responds to tactile stimuli; and 4- somnolent, responds to painful stimuli). Naloxone (1-2mcg/kg) IV was administered if the score ≥ 3. The total number of supplemental doses of tramadol consumed in 24 h was also recorded. At the end of the study, mothers were asked to rate their satisfaction with the pain management on a 3-point scale (1- dissatisfied, 2- satisfied, 3- highly satisfied).

The primary outcome was the time to first analgesic request and the secondary measures of outcome included number of doses of tramadol administered and pain, nausea, sedation & satisfaction scores.

The sample size was calculated based on results obtained from a pilot study conducted at our institute. The sample size was aimed to detect a 25% reduction in the number of doses of tramadol consumed [mean 1.588, standard deviation (SD) 0.795]. Assuming the power of the study at 80% and a clinical significance of 95%, a total of 125 subjects were required to detect this difference. To account for an attrition rate of 10%, we aimed to enrol 138 subjects into the study.

The results were analysed using Medcalc® version 17.9.4. Maternal and intra-operative characteristics were assessed using student’s *t*-test (two tailed, unequal variances) and chi square test as appropriate. Continuous data were assessed for normality using the Shapiro-Wilk test. Normally distributed data (represented as mean ± SD) were assessed using the student’s t-test (two-tailed, unequal variances) and non-normally distributed data [represented as median (IQR)] were assessed using the Mann-Whitney U-test. Ordinal data were represented as median & interquartile range (IQR) and assessed using the Mann-Whitney U-test. The time to first analgesic request was assessed using the log rank test. A *p*-value < 0.05 was considered significant.

## Results

**Fig. 1:**
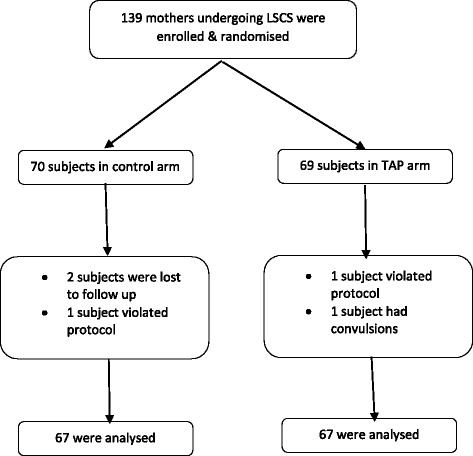
Flow diagram of randomisation and follow-up of enrolled participants. *LSCS* – lower segment caesarean section, *TAP* – transversus abdominis plane

A total of 139 mothers (70 in the control group and 69 in the study group) were enrolled into the study (Fig. 1). Two mothers in the control group were lost to follow up and were excluded from the study. One subject in each group violated protocol and had to be excluded from the study. One subject in the study group had convulsions following the injection of local anaesthetic solution. She developed convulsions in the PACU approximately 20 min after performing the block. She was managed conservatively with IV midazolam (2 mg) following which she recovered. She was closely monitored for the next 24 h during which no untoward events were noted. She also had to be excluded from the study.

**Fig. 2:**
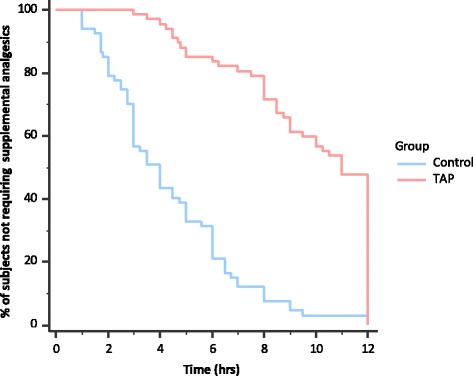
Kaplan Meier graph showing the % of patients in each group not requiring supplemental analgesia over time (*p* < 0.0001, log rank test). *TAP* – transversus abdominis plane

**Fig. 3:**
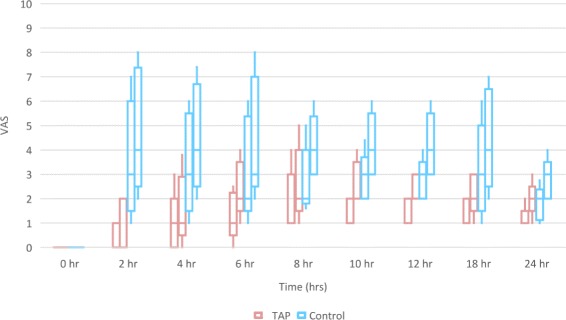
Box and plot graph of pain scores (VAS) over 24 h. At each time point, the first bar represents VAS scores of pain at rest in the study group; the second bar, VAS scores of pain on movement in the study group; the third bar, VAS scores of pain at rest in the control group; and the fourth bar, VAS scores of pain on movement in in the control group. The middle line in each box represents the median value, the outer margins of the box represents the interquartile range and the whiskers represent the 10th & 90th percentile at each time point (*p* < 0.0001, Mann-Whitney U-test). *VAS* – visual analog scale, *TAP* – transversus abdominis plane

Maternal and intra-operative characteristics were similar in both the groups (*p* > 0.05) (Table 1). The median (IQR) time to first analgesic request was 11 h (8,12) in the TAP group and 4 h (2.5,6) in the study group (Fig. 2). This difference was significant [*p* < 0.0001; 95% confidence interval (CI), 5.6 to 8)]. The median (IQR) number of tramadol doses consumed in the TAP group was 0 (0,1) compared to 2 (1,2) in the control group (*p* < 0.0001; 95% C.I., 1 to 2). At all points during the study, pain scores both at rest and on movement were significantly lower in the study groups compared to the placebo group (*p* < 0.0001, Fig. 3). Nausea scores were significantly lower (*p* < 0.05) in the study group only during the latter half (10,12,18 & 24 h) of the study. There was no difference with respect to sedation between the two groups. None of the subjects needed naloxone. The median (IQR) maternal satisfaction score was significantly higher in the TAP group compared to the control group; 2 (2,3) in the TAP group compared to 2 (2,2) in the control group (*p* 0.0002; 95% C.I., 0 to 1).

**Table 1:** Demographic variables & intra-operative characteristics of the study participants (*p* > 0.05, unpaired student’s *t*-test, Mann-Whitney U-test)

Characteristic	TAP Group (*n* = 67)	Control Group (*n* = 67)	*p* value
Age (years) (mean ± SD)	28.2 ± 4.7	28.4 ± 4.5	0.8114
Height (cm) (mean ± SD)	150.5 ± 4	150.6 ± 4.2	0.9422
Weight (kg) (mean ± SD)	64.5 ± 8.1	63 ± 6.8	0.2450
Parity (number/range)	1 (0-3)	1 (0-3)	0.3031
Gestational Age (weeks) (mean ± SD)	37.7 ± 1.9	37.6 ± 1.7	0.7405
ASA Status (II/III) (number)	63/4	63/4	1
Elective/Emergency (number)	36/31	42/25	0.577
Dose of 0.5% Bupivacaine (mg) (mean ± SD)	13 ± 0.5	12.8 ± 0.9	0.9568
Duration of anaesthesia (min) (mean ± SD)	69.8 ± 14.7	72 ± 17.2	0.4203
Duration of surgery (min) (mean ± SD)	57.8 ± 13.6	58.8 ± 15.6	0.6803

*TAP* transversus abdominis plane*, ASA* American society of anaesthesiologists

## Discussion

Post-operative analgesia after caesarean section is challenging since it should cater to maternal comfort and simultaneously have no adverse effects on the new born. Although neuraxial opioids provide good analgesia, they are associated with various adverse effects like nausea and pruritus which decrease overall patient satisfaction [1]. In addition, risks of delayed respiratory depression due to rostral spread of hydrophilic opioids like morphine [9] and adverse effects on the newborn [10] remain significant concerns. Therefore, techniques like TAP block have been employed to reduce opioid consumption and hence their adverse effects. TAP block as a component of multimodal analgesic regimen has considerable potential to improve the quality of pain relief and decrease analgesic requirements when used for analgesia after LSCS [5].

Our study demonstrated that supplementing a multimodal analgesic regimen with a TAP block reduces pain scores and additional analgesic requirements and hence their associated adverse effects. It also delays the time to first analgesic request and provides better satisfaction with pain relief compared with the standard regimen alone. Various trials comparing TAP block to a sham block have shown that TAP block produces superior analgesia, reduces supplemental opioid analgesic consumption and decreases the incidence of opioid induced adverse effects when used as a component of multimodal analgesic regimen for post caesarean analgesia [5, 11–13].

Since neuraxial morphine has been established as the best modality for post caesarean analgesia [9], various trials have compared the analgesic efficacy of TAP block with intrathecal morphine. They noted that superior analgesia was seen with intrathecal morphine as compared to TAP block but at the expense of adverse effects [14–16]. Moreover, trials that supplemented TAP block to an analgesic regimen inclusive of intrathecal morphine reported no additional benefits of adding TAP block to a regimen that includes intrathecal morphine [16–18].

Systematic reviews and meta-analyses also reported that TAP block produces superior analgesia and reduces supplemental opioid consumption when compared to placebo in the setting of multimodal analgesia for caesarean section that excluded intrathecal morphine. However, these benefits were difficult to demonstrate when intrathecal morphine was used. They concluded that TAP block could be considered as an alternative to intrathecal morphine wherever it is contraindicated or produces undesirable adverse effects [19–21].

The risk of complications following TAP block remains unknown. The subject weighing 60 kg received 150 mg ropivacaine (maximum permissible dose 3 mg/kg i.e. 180 mg). We suspect this to be a case of delayed absorption of the drug though the total dose of the drug was well within the permissible limits. Although ultrasound allows real time needle visualisation, it does not guarantee that the tip of the needle is in the plane and partial intramuscular or intraperitoneal injection may have occurred. We could not get blood levels of ropivacaine as the test was unavailable at our centre. To minimise such complications in the future, measures like decreased drug concentrations (0.3% ropivacaine instead of 0.375% ropivacaine), use of adrenaline to decrease systemic absorption, visualisation of the needle tip at all times during the procedure to prevent inadvertent intramuscular/intraperitoneal injection and acquiring lipid emulsion have been incorporated into the analgesia protocol.

Very few cases of complications with TAP block have been reported in literature so far [22, 23]. There are studies that have shown potentially toxic concentrations of local anaesthetics after TAP blocks [24–26]. In addition, pregnancy can predispose to local anaesthetic systemic toxicity (LAST). Various factors like the reduced dose of local anaesthetic that can cause convulsions [26], increased concentrations of free drug available due to decreased protein binding, increased venous distension of inferior vena cava (IVC) and an increased cardiac output leading to increased uptake and distribution of the drug [27, 28] and an increased neuronal susceptibility to local anaesthetics itself can predispose to LAST in pregnant mothers [27]. Finally, the concomitant use of subarachnoid block for caesarean section could also increase the systemic absorption of the drug due to vasodilation induced by sympathetic blockade thereby predisposing to systemic toxicity [22]. It would therefore be advisable to use the lowest possible concentration of local anaesthetic necessary to achieve the desired spread for a successful block.

Finally, while randomised controlled trials demonstrate the analgesic efficacy of TAP block, they are not largely powered to identify rare complications of the block. These trials do not require large sample population to demonstrate the correlation between treatment and effect as would be required to assess the safety of the block. Hence larger safety trials are needed. Additionally, a consensus needs to be developed regarding the safe dose and concentration of local anaesthetic solution to limit the systemic toxic complications of the block without affecting its analgesic efficacy.

Though our study identified areas for future research, it had certain limitations. The sample size of our trial was insufficient to assess the safety of the block. Also, the subjects were not followed up long term for the incidence of chronic pain. While all measures to conceal the group allocation were taken, true blinding may not have been possible since subjects in the study group reported less pain.

## Conclusion

We would like to conclude that TAP block reduces pain, prolongs the time to first analgesic request and decreases supplemental opioid analgesic requirement when used as a component of multimodal analgesic regimen for pain relief after caesarean section. However, the risk of local anaesthetic systemic toxicity remains unknown with this block. Hence larger safety trials and measures to limit this complication need to be ascertained.

## Abbreviations

BMI: Body Mass Index
CI: Confidence Interval
IQR: Interquartile Range
IV: Intravenous
IVC: Inferior Vena Cava
LAST: Local Anaesthetic Systemic Toxicity
LSCS: Lower Segment Caesarean Section
NSAIDs: Non-Steroidal Anti Inflammatory Drugs
PCA: Patient Controlled Analgesia
SD: Standard Deviation
TAP: Transversus Abdominis Plane
US: Ultrasound
VAS: Visual Analog Scale
